# Address

**Published:** 1887-09

**Authors:** E. H. Gregory

**Affiliations:** St. Louis


### Address.

BY DR. E. H. GREGORY, ST. LOUIS.

President of the American Medical Association.

Read before that Body, at the Meeting Held in Chicago, June, 1887.

Obviously, force and matter "make up" the universe. Force implies antagonism, antagonism perpetuates motion. The living cell is the embodiment of nature. Cell antagonism is life. Multicellular organisms represent a community of unities. A model organism in equillibrium is health. Cell struggle is the gist of modern pathology. Every organ and every element are vulnerable. The strength of resistance in elements and organs, reinforced by the harmony and precision of co-ordination and the vigor of counter agencies, are the momentous questions.

In seeking for a comprehensive title for my address, I fell upon the two simple words "Cell Antagonism," which form the foundation of my symptomatology and pathology, conjoined with cell changes, the basis of pathological anatomy, embracing at once the universe of life and all the possibilities of life; disease being but one of the multitudinous phases of life.

Huxley has likened the body to an army. "Of this army, each cell is a soldier, an organ, a brigade; the central nervous system, headquarters and field telegraph; the alimentary and circulatory system, the commissariat. Losses are made good by recruits born in the camp, and the life of the individual is a campaign, conducted successfully for a number of years, but with certain defeat in the long run." A model organism assumes that every soldier is a stalwart; every organ a body of stalwarts, with corresponding machines of co-ordination and precision, all alike concerted and vigorous to meet the myriads of counter forces, many of which are too cunning for our ken, but ever ready to take advantage of the least failure of our strength to penetrate our vitals. This force of vital resistance forms the basis of therapeutics, this "vis mediatrix naturæ" will be always the indispensable auxiliary of the physician, without which ally abandonment of the art would be inevitable.

The morphologist distinguishes two classes of elements within the organism. One class represents the fixed cells, cells which occupy the fortifications within the inter-cellular substance; the other class provides for the formation of new tissue, and represents the standing army, sometimes called embryonal substance. Before Cohnheim, our knowledge of the mobile elements—the mobolized army—of the body, was nothing; now most of the literature relating to neoplasm is or has become obsolete.. Now coagulable lymph is impossible without the white corpuscles. Organizable fluids have vanished. Through the ingenuity and industry of the great German pathologist a revolution has been worked in medical thought. The study of the mobile elements of the body—the relatively independent elements—promises much. We can almost see daybreak in pathology, a dawn before the coming light which is to illuminate some of the dark places of this most intricate subject. Already leucocytes substitute fibrin. The latter is the product of the former. Wherever there is localized impairment of nutrition, the result of inherent weakness, the leucocytes concentrate, feast on the devitalized structure, and thereby develop into a tissue replacing that which they have consumed. When the surgeon wishes to dispose of an adventitions structure—a cyst, for example—in the connective tissue, he lowers the vitality by over-stimulation—irritation—when he confidently expects the leucocytes to congregate and destroy the cyst, substituting a granulation tissue, which in time becomes scar tissue. Now an organizable fluid as the essential product of inflammation, cells playing a passive part, is a thing of the past. Now cell potency, cell subtlety, and cell antagonism constitute the pith and marrow of medical science. There was a period in the history of pathology when all the neoplasia sprang from the tissue cells; now the place and destiny of the tissue cell is fixed. When the tissue elements yet contain some undifferentiated protoplasm, proliferation is possible, otherwise not probable. The mobile cells constitute the mobilized army; the great co-ordinating centers assemble them at any moment, ready to antagonize hurtful agencies. Irritation always provokes a concentration of the white cell. Irritation means injury. It may be simple or specific. Either way it is a signal for moving the army in solid phalanx to confront noxious agencies, and the combat is hotly contested till victory or discomfiture ensues.

Inflammation, the keystone of medical science, the standard process by which all other pathological conditions are measured, is only to be explained on the theory of cell antagonism; it is practically a struggle between irritant bodies and white blood corpuscles. Foreign bodies are invariably invaded by leucocytes, their swarming having for its object to repel or render inert the offending cause. Observations directed to the white cell have been followed by an inspiring increase in our knowledge of the vital properties of cells in general. The quality of spontaneous motion once accepted, the idea that the cell played a passive part quickly disappeared, and in its stead came the conception of autonomy, the fact that the cell specialized food for its own purposes, actually breaking up compounds and adapting the the products to its own growth and development, strikingly illustrated by an independent form of government—for example, the government of the United States. A great statesman has said ours is a "government of the people, by the people, and for the people." Cells live of themselves, by themselves, and for the whole body. The cells make the central government. The co-ordinate machinery is formed by the cells, and the cells are in their turn centralized and harmonized by this power. The tissue elements are in direct continuity with the centre; but it is otherwise with the floating, wandering cells; yet their behavior gives evidence of their concern for the well-being of the entire organism. Again, the free cells take into their substance the most refractory substances, digesting solid bodied; sponges and green plants yielding to their digestive and assimilative processes. Observations the most patient and trustworthy in the lower vegetables have demonstrated the fact that bacteria have been seen in the substance of amœboid cells; others have been seen pursuing bacteria, ultimately capturing and destroying them. Throughout the whole animal kingdom mesoderm cells use their ingestive power for destroying micro-organisms. Further, this property seems to be utilized for the removal of larv organs. Colorless corpuscles present the same appearance and have similar properties and the same mode of origin in the entire range of living beings. When the irritant or harmful agency is particularly obdurate, white cells exhibit a very strange habit of throwing out processes which unite with similar processes from neighboring cells, until a considerable mass of protoplasm is formed by their confluence, constituting a giant cell. Thus an army of giants may be improvised on occasions of extreme emergency; for example, when the tail and gills of the tadpole are to be swept off, this powerful division is ready for the Herculean task.

Apply these facts to the inflammatory process in mammals. Suppose a foreign body lodged; that moment leucocytes leave the blood vessels and congregate for the purpose of opposing the intruder. In such diseases as tubercle, leprosy, etc., the giant cells appear, and in their center—their substance—the specific bacillus is found. Koch found the bacillus of anthrasis and the bacillus of septacæmia in the mouse, enclosed by white blood cells. We say, with these observations before us, inflammation exhibits a new aspect; cells conquering cells as a process of normal physiology; simply the free cells, the white cells resisting injury, there being a provision not only for concentration, but for increasing their number and augmenting their strength, corresponding to the emergency. A recent authority, Mr. Sutton, says: "If we summarize the story of inflammation as we read it zoologically, it should be likened to a battle. The leucocytes are the defending army; their roads and lines of communication the blood-vessels. Every composite organism maintains a certain proportion of leucocytes as representing its standing army. When the body is invaded by bacilli, bacteria, micrococci, chemical or other irritants, information of the agression is telegraphed by means of the vaso-motor nerves, and the leucocytes rush to the attack, reinforcements and recruits are quickly found to increase the standing army, sometimes twenty, thirty, or forty times the normal standard. In the conflict cells die and often are eaten by their companions; frequently the slaughter is so great the tissues become burdened by the dead bodies of soldiers in the form of pus, the activity of the cells being testified by the fact the protoplasm often contains bacilli, etc., in various stages of destruction. The dead cells, like the corpses of soldiers that fall in battle, later become hurtful to the organism they in their lifetime were anxions to protect from harm, for they are fertile sources of septicæmia and pyæmia, the pestilence and scourge so much dreaded by operative surgeons. The analogy may seem to some a little romantic, but it appears to be warranted by the facts.

Just here the question obtrudes: Is the action which follows mechanical, chemical and thermal injuries, and the action caused by vital injuries alike inflammatory processes? In the one, that resulting from physical causes, the nutrient processes are not disturbed, simply increased. In the other, that resulting from vital causes, the cell processes are disturbed, thwarted, and vitiated. It seems unfortunate that the action which succeeds to traumatism should be confounded with that which succeeds to specific germs. The nutrient changes come into vigorous play after an ordinary injury, altered in one particular only, viz.: the presence of palpable neoplasm, which substitutes the structures doomed by the injury. The addition of new material is invisible in ordinary nutrition, growth and maintenance for example, the substitution being atomic. When a structure is destroyed bodily, as in traumatism, the substitution is corpuscular, therefore palpable. It is certainly perplexing to assume, as does Sir James Paget, that a visible neoplasm is produced by inflammation, but its development is impossible till after the withdrawal of the inflammation. To get rid of confusion, though we do not emerge from error, is to make a step toward truth. The full effect of traumatism is immediate and complete ; it cannot increase itself, therefore its effects are always limited and within the possibility of estimation, for the reason that cause and effects are in precise correspondence ; besides antiseptics do not influence the changes in any particular. On the other hand, the most extensive and severe injuries of vascular parts, involving bone, joints, the great cavities, etc., is possible without producing inflammation, provided antiseptic precautions be taken. When such complication ensues, it is a wound accident, not a wound incident, and the result of infection. There is no disturbing factor in the process incident to repair; no waste products. If pus is found, it is the result of the intrusion of some noxious agency, some contagion, some pestilence; in short, a specific cause.

If it be agreed to call the action inseparable from ordinary injury physiological repair, the term inflammation is limited to the series of events referable to a vital agency, which until very recently has eluded detection. A specific agency determines a class of diseases as distinctive and definite as the living creatures studied in natural history. Cell antagonism after physical injury is simply a substitution, through the development of mobile cells, of the tissue which has been spoiled. The fixed elements multiply and develop in the expansion and perfection of the several structures of which they are components, but take no active part in the process of repair after injury. After the perfection of the body, the factors favoring proliferation and those which inhabit it must be in a state of balance. In functional hypertrophy, the balance is toward the side of proliferation. On the other hand, in the non-functional hypertrophy, the problem is not so simple; the balance is disturbed toward proliferation by some mysterious agency. How strikingly different when the cell antagonism is with any vital injury; here cell mysteries clash. The bacteriæ enter into conflict with mobile and fixed cells, but it is not possible to know how the conflict is carried on. Certainly a series of disturbances ensue in the normal metabolism of the elements. The functional, formative and nutritive activities, which are the expressions of cell life, must be altered; their vigor, perhaps, lessened, their susceptibilities modified. Exceptionally their life is enfeebled or extinguished. The issue of a bacterial affection is either the death of the patient or the destruction and elimination of the bacteriæ. It follows that this disturbance of cellular activities is always at the bottom of morbid symptomatology; and observations have shown that disease of this kind, successfully withstood, leaves the element in a peculiar unsusceptible condition, insuring an immunity, almost or quite complete, against a fresh invasion of the same or kindred bacteriæ. This modified susceptibility was practically understood by Jenner. Pasteur, resuming and systematizing the great Englishman's work, successfully modified cell forces, rendering them harmless by cultivation, and sending them on the important mission of destroying the natural proneness to the deadly assaults of the uncultured cells. Whilst we may despair of ever understanding the essential nature of vitality, the study of the causes which regulate life, and their subordination to conditions which may be determined, has led the way to the grandest achievements of recent times. Pasteur and Lister are the great apostles of modern practical thought.

Huxley has suggested that it may be possible to introduce into the economy a molecular mechanism which, like a cunningly contrived torpedo, shall find its way to some particular group of cells and cause an explosion among them, leaving the rest untouched. Pasteur's cell mechanisms exceed the conception of the great scientist, for they cunningly change the vital qualities of the elements without destroying them. May we not look forward for some great advance in therapeutics in the direction to which Pasteur's genius points, by the study of cell antagonism? Certainly vital subtleties may best cope with vital subtleties. Study of the conditions in which infective agents arise, by ascertaining the circumstances which limit or facilitate their diffusion, has already raised surgery to a proud pre-eminence. With therapeutics it is otherwise, as it is doubtful whether the facts have yielded the slightest increase of power against the diseases of mankind. Let us not despair. Knowledge must come first, then wisdom brings its practical application. The study of cell possibilities, their readiness and energy in rendering inert noxious agencies, whether introduced from without or arising from within, exhibiting an antagonism at once potent and direct, rather tends to dampen one's therapeutic enthusiasm. The thoughtful student sees nothing abnormal in disease. To be sure there is a physiological emergency, but there is no disorder in the cell processes ; rather the perfection of order. If a physical or chemical cause intensifies the cell changes, in other words, determines the physiological emergency—every cell movement is direct, purposive, and efficient, ending only when the intruder is ejected, encysted, or accommodated. On the other hand, if the offense be a vital one, a living cell, a microphite, the spectacle is that of one living creature preying upon another, the declaration of the first law of nature, not an enemy, but an intruder, struggling for self-preservation, simply a physiological fight for life. Can we hope for an ideal tonic for cell antagonism, such as would innervate the cells on one side, and enervate them on the other, the "old chestnut," "Prevention is better than cure," is not yet worm eaten.

Cell antagonism implies a struggle. The duration of the struggle is determined by the quality of the irritant and the strength and resources of the antagonist. Acute inflammation is a sharp and decisive action, chronic inflammation slow and indefinite. There are two striking important chronic conditions, viz: The interstitial inflammation and the infective granulomata, interesting alike to the physician and the surgeon; both are alike disastrous, the one, interstitial inflammation destroying by cicatrical contraction; the other, infective granulomato, by stopping at the fibro-blast stage, retrogression replacing development. The quality of infection and the failure to develop beyond the stage of granulative tissue is the exact condition, as illustrated by tuberculosis, syphilis, lupus, etc., in contrast with which is fibrosis of the liver, cirrhosis of the kidney, Bright's disease, fibrosis of the brain, sclerosis, interstitial inflammation, destroying the tissue elements by strangulation and infective granulomata by contamination.

Again, the two varieties of inflammation referred to declare that the quality of the irritant determines the effect of the inflammation, the interstitial variety being caused by physical agency, and the infective by a vital one. Cells antagonize physical causes without losing any of their vital qualities. Not so when cells antagonize vital causes. When cells are pitted against cells they may be despoiled of their highest quality, as in the infection granulomata they have parted with formative activity. In short, the knowledge that pertains to the presence of a vital irritant epitomizes all that has been taught of infective disorders. The symptomatology and pathology of this class of diseases is but the life history, the play of cell activities, metabolisms, and catalysis of antagonizing organisms, as likewise to the knowledge pertaining to the presence of a foreign body or physical irritant is the knowledge, in brief, of fibrinoses in general of that entire class of disorders known as interstitional inflammation. A persistant irritant—injury—with its attendant concentration of leucocytes, their inevitable development and ultimate transformation, the history of the capsulation of a foreign body, whether it be animate or inanimate is the history of cirrhosis of the liver, fibrosis of the lung, morbus Brightii, atrophy of the heart, atrophy of voluntary muscles in general, sclerosis of the brain and bones; in short, diffused capsulation. Some disseminated irritant, say alcohol, determines a sclerosis of the liver, and how? Every particle of alcohol being a foreign particle, having its capsule, it follows that capsulation is as uniformly distributed as the cause. An artisan inhales fine particles ot steel or other foreign material, and fibrinosis of the lung is the consequence. The irritants of gout and rheumatism are lodged in the kidney, the interstitial tissue of the organ increases, and in the end strangulates the tubules and malpighian bodies, resulting in shrinking and total disorganization of the organ, constituting Bright's disease. Like changes occur in the heart co-incident with the development of the interstitial fibrous tissue as atrophy of the muscular substance, substituting a fibrous induration for its normal structure. A similar change in the voluntary muscles occurs in the curious disease known as the pseudo-hypertrophic paralysis, chiefly afflicting children. The connective tissue between the muscular fibres increases so much that the muscles affected may exceed their normal size three times. Later, however, the new tissue shrinks, and the contractile material of the muscle is spoiled. The condition of sclerosis in bone corresponds to cirrhosis of the liver, and Bright's disease of the kidney. In the nerve centers of the interstitial tissue, neuralgia takes on the same chronic over-growth, strangling the nerve strands and cells, giving rise to the most singular and complicated nervous phenomena. The changes thus induced are recognized by the general term sclerosis. If it involves the fascicules of Burdack and the column of Goll, locomotor ataxia results, if the medulla, bulboo paralysis, etc.

We have purposely avoided reference to diathesis, as also to the precise neurological relationship of cells, not because we deny their influence, but because too little is definitely known. On the contrary the behavior of cells relatively to irritants is well understood. The presence of a foreign body is at once and directly resisted by the leucocytes, a zone of inflammation, its destruction, transportation, or capsulation, even parasites which resist the death-dealing assaults of the leucocytes are at last imprisoned in cases of fibrous tissue.

There yet remains for our consideration a mysterious possibility relating to cell life of great practical moment, viz.: that of entering upon a life of independence, separating from the central nervous system "headquarters," disregarding the "field telegraph," oblivious to the morning "drumbeat," and wholly the restraining and directing influence of environing structures. We have learned that however much irritation may affect the vital qualities of cells, their fidelity is always preserved, there being no sign of disaffection, the army ever intact, the fealty of the soldiers supreme. Physical causes—traumatism—may crush or impair the tissues, but the cells come to the rescue with all vigor and directness. The prolonged presence of bacteriæ may impair the forces of the cells, enfeeble and extinguish them, but as long as life lasts their efforts are in harmony with the purposes of the organism. Thus it appears that alienation is not possible through the agency of irritation, however intensified, modified, or prolonged.

The question constantly obtrudes: Is alienation possible to a mature element, to an element that has assimilated itself physiologically as well as anatomically, with the surrounding tissues; taking part in its functions; concurring and co-operating in all the processes of the economy? This question seems to have confronted Cohnheim when his ingenuity suggested the embryonal hypothesis, viz.: that the tumor germ was congenital; that there were in the mature body embryonal elements and tissues not utilized in the elaboration of the normal structures; latent, embryonal rudiments; small embryonal cells so diminutive as to elude observation, inactive perhaps, till some exciting cause awakens activity. Is not an embryonic element in a mature organism an alien? Is it not already an independent element? Are not the patches in the skin, pigmented moles, islands of cartilage in mature bone, congenital angloma, etc., independent structures, already tumor germs, congenital rudiments of tumors? Accept the hypothesis of the great German pathologist, and the problem of tumor etiology is almost solved; it remains only to find the exciting cause; the predisposition is inherited; irritation, simple or otherwise, may be nearly allied to the awakening impulse. The difficulty is to reconcile cell antagonism with the proposition this doctrine includes; the idea that all discordant bodies are treated as intruders, either ingested, transported, or covered up. We can conceive the possibility of the existence of an inoffensive tissue of element, one that simply draws its nourishment from adjacent tissue, without any serious disturbance of their normal metabolism. We would gladly adopt the theory of Cohnheim, which ascribes the origin of tumor neoplasms to persistent germinal rudiment. Certainly it has much to recommend it. Countless latent embryonal structures, relatively independent, are tolerated by the organism, remaining inactive till favored by some concurrent event, the nature of which is incomprehensible, when they may grow according to their bent. Is it not quite as possible there may be similar, relatively independent elements, ready when stimulated, to multiply independently? Congenital angloma often grow without definite limit; pigmented moles; black, slightly-raised patches in the skin, with which all are familiar, are composed of tissue exactly resembling sarcoma, always suspicious because of their disposition to proliferate independently. It is most difficult to believe in the infidelity of covenant cells, of sundering the compact which unifies the organism. Emancipation seems almost out of question. We must not only believe that dependent benignant elements become the elements of a parasite—a parasite so vigorous, corrupt, and wicked as to destroy its source.

